# 

**Published:** 1898-08-20

**Authors:** 


					THE HOSPITAL. " The standard of highest purity."?Lancet, August 20th, 1898.
W CADBURY'S In CADBURY'S
-\
COOOA
ABSOLUTELY PURE. Jb ^ ^ NO DRUGS OR ALKALIES USED.
COCOA
'R wich hospital
I NrIRMARY
No. 621. Vol. XXIV. Sattjbday,
August 20th, 1898.
'8ubSS3Pp?3Eo!rn:8'] PRICE TWOPENCE.

				

